# Study of Radiation-Induced Damage Processes in CeZrO_4_–YZrO_3_ Ceramics Caused by Helium Irradiation

**DOI:** 10.3390/ma16010198

**Published:** 2022-12-26

**Authors:** Artem Kozlovskiy, Daryn B. Borgekov, Maxim V. Zdorovets, Kayrat K. Kadyrzhanov, Dmitriy I. Shlimas

**Affiliations:** 1 Laboratory of Solid State Physics, The Institute of Nuclear Physics, Almaty 050032, Kazakhstan; 2 Engineering Profile Laboratory, L.N. Gumilyov Eurasian National University, Astana 010008, Kazakhstan

**Keywords:** inert matrices of nuclear fuel, composite ceramics, zirconium dioxide, radiation defects, structural disorder

## Abstract

Composite oxide ceramics CeZrO_4_–YZrO_3_ obtained by mechanochemical synthesis were chosen as objects of study. The most dangerous type of radiation defect in structural materials is associated with helium accumulation in the structure of the near-surface layer. This can lead to the destruction and swelling of the material, resulting in a decrease in its strength and thermal characteristics. During the studies, it was found that the most significant structural changes (deformation of the crystal lattice, the magnitude of microdistortions of the crystal lattice) are observed with irradiation fluence above 5×10^16^ ion/cm^2^, while the nature of the changes is exponential. X-ray diffraction analysis found that the nature of the crystal structure deformation has a pronounced type of stretching due to the accumulation of implanted helium and its subsequent agglomeration. A comparative analysis with data on microdistortions of the crystal lattice and the values of microhardness and softening of ZrO_2_ and CeO_2_ showed that two-phase ceramics of the cubic type CeZrO_4_-YZrO_3_ are more resistant to radiation-induced degradation than single-phase ZrO_2_ and CeO_2_. Results of strength and thermophysical characteristics showed that the presence of two phases increases resistance to destruction and disorder, leading to a decrease in strength and thermal conductivity.

## 1. Introduction

Over the past few years, the nuclear power industry has been actively considering the possibility of switching from traditional nuclear fuel based on uranium dioxide to dispersed nuclear fuel, in which plutonium (usually weapons-grade) is placed in an inert matrix, serving mainly as an absorber of fission fragments, as well as products of nuclear reactions during the interaction of fissile fuel with neutrons acts as the primary fissile material [1,2,3]. Interest in this type of fuel cells or fuel assemblies is primarily due to the possibility of using stocks of weapons-grade plutonium for peaceful energy production and reducing the concentration of fission products and nuclear waste in the production of electricity [4,5]. At the same time, the use of such a type of fuel makes it possible to expand the potential of using nuclear fuel by increasing the burnup efficiency and the operating temperature of the core, as well as increasing the service life of fuel elements, which are due to their radiation damage accumulation resistance [6,7].

One of the key problems in the nuclear power industry when using various types of structural materials, both traditional and new types based on high-temperature oxide, nitride or carbide ceramics, is the problem of gas swelling, which can arise under the radiation impact of both uranium fission fragments or neutron irradiation and the nuclear reactions caused by them, and during interactions with coolants [8,9,10]. As a rule, gas swelling processes are long-term processes accompanied by a long-term accumulation of radiation damage or implanted helium, xenon, or krypton ions, followed by the formation and filling of cavities or pores, forming gas-filled bubbles [11,12,13]. With an increase in the concentration of gases in the bubbles, an increase in the internal pressure occurs, which in turn leads to expansion and an increase in the volume of the bubble; due to the achievement of critical pressure, their rupture occurs, accompanied by the destruction of the surface layer and the destruction of the material [14,15]. At the same time, in most cases, this problem is acute for materials in which helium accumulates, which have high mobility and the ability to agglomerate, as well as concentrate in a small surface layer (no more than 1–2 microns), can seriously disrupt the stability of the material, as well as to reduce its thermophysical parameters [16,17]. Unlike metals, for which the processes of helium swelling are well studied, in ceramic materials, due to their structural features, as well as the high resistance of the crystal lattice to external influences, the processes of degradation during the accumulation of helium and the consequences caused by its evolution have not been fully studied. However, these materials have excellent prospects for use in nuclear energy of the new generation [18,19].

At the same time, in the last few years, the use of composite oxide ceramics as a basis for inert matrix materials, which have higher strength and stability indicators than simple oxides, has been actively studied [20,21,22]. One of such promising materials is ceramics, which contains compounds of zirconium dioxide, cerium and yttrium, the combination of which gives ceramics high stability and thermal conductivity compared to pure zirconium dioxide, which is one of the candidate materials for nuclear power, and cerium and yttrium elements serve in this composite as protectors that increase the strength of ceramics [23,24,25].

To date, a fairly large number of works are devoted to studying the radiation resistance of oxide ceramics intended for use as materials for inert matrices of nuclear fuel [26,27,28]. Interest in this topic consists in the fact that, unlike metals, the processes of radiation damage and their accumulation in dielectric ceramics are currently poorly understood, and there is currently no general theory that describes the mechanisms and kinetics of radiation damage. For example, several works [29,30] showed that the processes of radiation damage in ZrO_2_ ceramics could lead to the initialization of polymorphic transformation processes, which are accompanied by a decrease in strength and thermal characteristics. At the same time, one of the ways to combat these processes is the stabilization of zirconium ceramics with yttrium or cerium, the addition of which leads to an increase in resistance to radiation damage [31,32,33]. In addition, several works [34,35,36] devoted to studying the radiation resistance of CeO_2_ ceramics have shown rather excellent prospects for these materials when used as a basis for creating dispersed nuclear fuel. However, despite the sufficient amount of experimental work in this area, many issues still need to be addressed. One of these issues is assessing the resistance of double ceramics of the CeZrO_4_–YZrO_3_ type to radiation damage processes and changes in their thermophysical and strength properties. Interest in these types of ceramics, as well as the study of their radiation damage resistance, is due to the prospects for their use as a basis for inert matrices of dispersed nuclear fuel and structural materials for nuclear reactors. At the same time, the interest in the study of the radiation damage mechanisms associated with the implanted helium accumulation in the surface layer is due to the possibility of obtaining new data on the kinetics of radiation defects and their evolution in oxide ceramics. There is also interest relating to testing the hypotheses about the phase composition effect—in particular, the presence of two phases on the resistance to radiation destruction and deterioration of the properties of ceramics.

## 2. Materials and Methods

The synthesis of CeZrO_4_–YZrO_3_ ceramics was carried out by solid-phase mechanochemical synthesis. The planetary mill, Pulverisette 6 classic line (Fritsch, Berlin, Germany), was used for grinding. To obtain the CeZrO_4_–YZrO_3_ ceramics, we used initial powders of ZrO_2_, CeO_2_, and Y_2_O_3_ oxides at a concentration of ZrO_2_:CeO_2_:Y_2_O_3_ equal to 0.45:0.45:0.10 mol. All powders were purchased from Sigma Aldrich (Sigma Aldrich, Burlington, MA, USA), and the chemical purity of the powders was 99.95%. After grinding, the samples were pressed into tablets 0.05 mm thick and 8 mm in diameter for further studies related to irradiation and measurement of material properties. After grinding the initial mixtures at a grinding speed of 400 rpm for 1 h, the resulting mixtures were annealed at a temperature of 1500 °C for 5 h, followed by cooling with the furnace for 24 h. X-ray phase analysis on samples after thermal annealing was carried out to determine the phase formation and to establish the structural parameters. In addition, for verification, an X-ray phase analysis of the samples after mechanochemical milling was performed. It showed that the samples are a mixture of two phases of ZrO_2_ and CeO_2_ with a highly disordered structure associated with milling processes. Thus, it was determined that the main phase formation processes occur as a result of the thermal annealing of the samples.

To simulate the processes of radiation-induced degradation as a result of the accumulation of implanted helium in the near-surface layer with subsequent destruction and swelling, the Irradiation of samples was implemented on a DC-60 heavy ion accelerator (Institute of Nuclear Physics, Astana, Kazakhstan). Low-energy helium ions (He^2+^) with an energy of 40 keV were chosen for irradiation. Irradiation fluences were 10^14^–5 × 10^17^ ion/cm^2^; particle flux density was 10^9^ ion/cm^2^×s. Under the chosen irradiation conditions and the flux of particles, the effect of local overheating of the near-surface layer does not occur during irradiation. According to the calculated data of He^2+^ particles in the selected materials for irradiation, the maximum path length was 200–250 nm, and the energy losses were dE/dx_nuclear_ = 7 keV/µm, dE/dx_electron_ = 157 keV/µm. According to the estimate of energy losses, the main contribution to changes in the properties of materials during the interaction of incident particles with the crystal structure is made by electronic interactions over most of the path length. 

**Figure 1:**
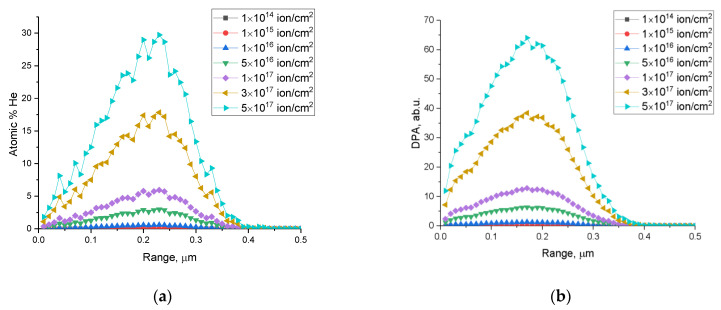
Calculation results for the dependences of (**a**) the concentration of implanted He^2+^ and (**b**) atomic displacements along the trajectory of incident particles for various irradiation fluences.

Figure 1 shows the calculated values of the concentration of implanted helium along the trajectory of incident ions in the ceramic material and the values of atomic displacements (*dpa*) that characterize the degree of radiation damage in the material. These results were built based on calculations performed in the SRIM Pro 2013 program code, using the Kinchin–Pease model, considering cascade effects that can occur during the interaction of incident ions with the target substance. The threshold displacement energy (*E_d_*) equal to 28 eV, 28 eV, and 33 eV for O, Ce, and Zr, respectively, was used for calculations.

The general form of the dependences presented indicates that the most significant radiation damage degree accumulates at a depth of 150–250 nm, and for maximum radiation fluences, the displacement value is 30–60 dpa, which is typical for serious doses of accumulated radiation damage in the near-surface layer, which can lead to destruction and disorder of the structure. According to the literature data of previous studies [37,38,39], the processes of destruction of the near-surface layer associated with the accumulation of implanted helium with the subsequent formation of helium bubbles, as a rule, are observed at fluences above 5 × 10^16^–10^17^ ions/cm^2^. At the same time, in most cases, critical doses leading to destruction and embrittlement are considered to be 3 × 10^17^–10^17^ ions/cm^2^. In the work of Egeland, G. W. et al. [40], a method for calculating the values of atomic displacements and the concentration of implanted helium in the near-surface layer of ceramics is given, which was used to calculate and build the dependencies shown in Figure 1.

As a rule, the value of atomic displacements (dpa) is used for a comparative analysis of various types of irradiation with neutron irradiation and characterizes the general trend of changes in radiation damage in the material. The presented data of dpa values in Figure 1 reflect the accumulation of radiation damage in the near-surface layer depending on the irradiation fluence, which can be compared with neutron exposure if necessary.

The study of the irradiation effect on the change in the structural features of the synthesized ceramics, as well as the determination of the nature of deformation distortions in the damaged layer, was carried out using the X-ray diffraction method, which was implemented on a D8 Advance ECO powder diffractometer (Bruker, Berlin, Germany). X-ray diffraction patterns were taken in the Bragg–Brentano geometry in the angular range 2θ = 25–85°. DiffracEVA v.4.2 software was used to interpret the obtained diffractograms. X-ray diffraction patterns were taken on samples pressed into pellets 0.05 mm thick and 8 mm in diameter. In this case, the conditions for recording X-ray diffraction patterns (current strength and voltage) were selected so that the diffraction patterns reflected changes in the damaged layer. In addition, the main changes were assessed by a comparative analysis of samples before and after irradiation, which made it possible to obtain data on changes in structural and thermophysical parameters due to the accumulation of radiation damage during irradiation.

The strength properties, particularly the microhardness of the near-surface layer and crack resistance, were determined using methods of indentation and single compression of samples.

The determination of thermophysical parameters, thermal conductivity coefficient and heat losses were carried out using the KIT-800 device by changing the longitudinal heat flow in the temperature range from 100 to 80 °C. Thermophysical parameters (thermal conductivity and heat loss) were measured on samples pressed into tablets, 0.05 mm thick and 8 mm in diameter. The measurements were carried out on the same samples before and after irradiation.

## 3. Results

**Figure 2:**
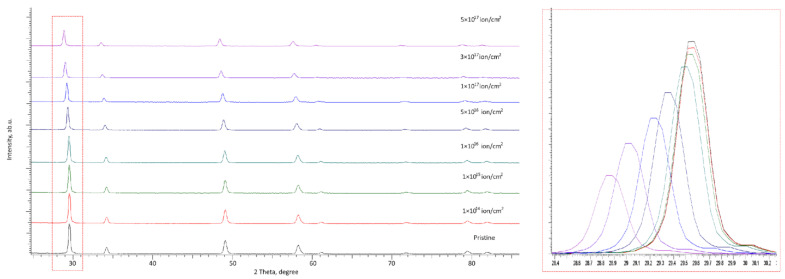
X-ray diffraction patterns of the studied samples depending on the irradiation fluence (the highlighted area reflects changes in the position and shape of the diffraction reflection at 2θ = 29–30°).

Figure 2 shows the X-ray diffraction results, reflecting changes in the structural characteristics of the studied ceramic samples depending on the fluence of irradiation with He^2+^ ions. For an example against which the changes caused by irradiation were compared, the X-ray diffraction pattern of the samples in the initial non-irradiated state is given. According to the analysis of the obtained diffraction pattern, the studied samples are a compound of two CeZrO_4_ and YZrO_3_ cubic phases, the main diffraction lines of which are quite close to each other. The phases were determined using the decomposition of diffraction patterns by the Rietveld method, followed by refinement of the structural parameters.

According to the data presented, when the irradiation conditions change, in particular, an increase in the irradiation fluence, the main changes caused by irradiation are a change in the shape of diffraction reflections, as well as their shift and change in intensity, which indicates the deformation nature of distortions and their cumulative effect with fluence increase. In turn, the nature of the change in the position of the lines and the change in intensity indicates the formation of tensile deformation stresses in the structure, leading to a shift in the position of the maxima to the region of small angles. At the same time, the nature of this displacement has a pronounced dependence on the irradiation fluence and the accumulated dose of atomic displacements.

At the same time, it should be noted that the formation of new reflections in all presented X-ray diffraction patterns was not established, which indicates the absence of processes of initialization of polymorphic transformations and phase transformations in the structure of ceramics under the action of irradiation in the entire range of radiation doses and accumulated radiation damage. The absence of such changes indicates an increased resistance of the synthesized ceramics to changes characteristic of ceramics based on zirconium dioxide [29,30] associated with polymorphic transformations of the t-ZrO_2_ → c-ZrO_2_ type.

At low irradiation fluences of 10^14^–10^16^ ion/cm^2^, the main changes in diffraction reflections (according to the detailed representation of the change in the shape and position of the diffraction reflection at 2θ = 29–30°) are associated with a slight decrease in the intensity of reflections, and at a fluence above 10^15^ ion/cm^2^, a small shift to the region of small angles, which indicates the initialization of the radiation damage accumulation stage and its deformation effect on crystal structure distortions. In this case, small changes in the intensity of diffraction reflections are characteristic of the formation of structurally disordered regions in the damaged layer, the formation of which is associated with an increase in the implanted helium concentration and the atomic displacements caused by it.

With an increase in the irradiation fluence above 10^16^ ion/cm^2^, which is characterized by a sharp increase in the value of atomic displacements, the change in the intensity and shape of diffraction reflections become more pronounced and are characterized by a sharp decrease in intensity by more than 1.5–2 times in comparison with the initial values, as well as a large shift to the region of small angles, which is characteristic of an increase in the tensile strain contribution. Such a substantial deviation from the initial position of diffraction reflections indicates that when radiation damage accumulates in the structure, many deformation distortions are formed. These deformation distortions can be associated with the knocking out of atoms from the lattice sites and with the agglomeration of implanted helium. This leads to the formation of gas-filled cavities, followed by extrusion to the surface.

**Figure 3:**
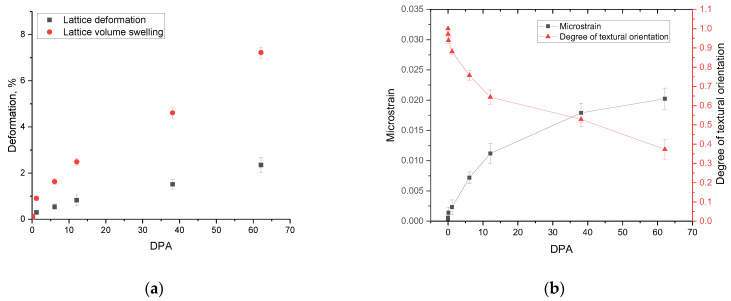
(**a**) Evaluation results of the crystal lattice deformation and swelling; (**b**) results of changes in microstresses and textural orientation as a function of the dose of atomic displacements.

The dynamics of changes in the crystal lattice deformation and an increase in its volume as a result of swelling are shown in Figure 3a, which reflects the structural distortion degree during the radiation damage accumulation. The data are presented depending on the magnitude of atomic displacements calculated based on the simulation results. As can be seen from the data presented, with an increase in the irradiation fluence, as a consequence, the value of atomic displacements and structural distortions grow exponentially. According to the data obtained from the analysis of changes in structural parameters, the deformation distortion degree changes insignificantly with an increase in the value of atomic displacements at high fluences. This behavior may be due to the effect of the accumulation of structural distortions with increasing fluence. The effect is because, at high fluences, the structure is distorted to such an extent that its further deformation can be hindered due to distortions that prevent their further propagation.

In the case of a change in the swelling value at high irradiation fluences, the swelling value increases more than the value of structural distortions. The difference can be explained by the fact that, at high irradiation fluences, the implanted helium begins to heat up in the structure and subsequently agglomerate. This is followed by an increase in the structure volume due to the filling of cavities with helium, which are formed due to crystal structure distortions and the displacement of atoms. Thus, it can be concluded that at fluences above 10^17^ ion/cm^2^, the main contribution to the destruction of the near-surface layer is made by the effects associated with swelling due to the implanted helium accumulation and subsequent agglomeration.

Figure 3b shows the results of the changes in the value of microstresses and texture orientation of the studied ceramic samples depending on the value of the accumulated dose of atomic displacements. The value of microstresses was calculated based on changes in the interplanar distance displacement value, for the main observed diffraction reflections, reflecting the nature of the volumetric distortion of the structure. The textural orientation degree was estimated by comparing the intensities of reflections before and after irradiation depending on the accumulated irradiation dose and reflecting the misorientation of crystallites in the material under external influences.

As can be seen from the data presented, the value of changes in microdistortions, as in the case of crystal lattice deformation, has a saturation effect, which was previously reported in several works [41,42] related to the description of radiation damage in ceramics and metals. This behavior can be because with an increase in the value of atomic displacements, the formation of cluster defects reported in [30] occurs, and deformation distortions of the structure, which do not give further acceleration and migration of displaced atoms due to their clustering.

During analysis of the texture misorientation depending on the radiation fluence and the value of atomic displacements, it is evident that at small displacements, misorientation is practically not observed (its value is less than 3%). However, if the displacement value is more than 1 dpa, the texture is misoriented, and at large displacements, there is a complete misorientation and the absence of a preferred texture direction.

One of the important indicators of the applicability of inert matrix materials under prolonged exposure to radiation is their destruction resistance and the preservation of strength characteristics, including hardness and crack resistance. A change in the strength properties of ceramics due to irradiation and the subsequent accumulation of radiation damage can lead to embrittlement of the material of the inert matrix, leading to the destabilization of nuclear fuel. According to the main requirement for dispersed nuclear fuel concerning the preservation of long-term stability of the inert matrix strength, as a result of the radiation damage accumulation, it is necessary that the fissile material of the nuclear fuel is constantly surrounded by an inert matrix capable of absorbing fission fragments and products of nuclear reactions. While the volume of the inert matrix material, which retains its performance close to the initial values, must significantly exceed the thickness of the damaged layer.

**Figure 4:**
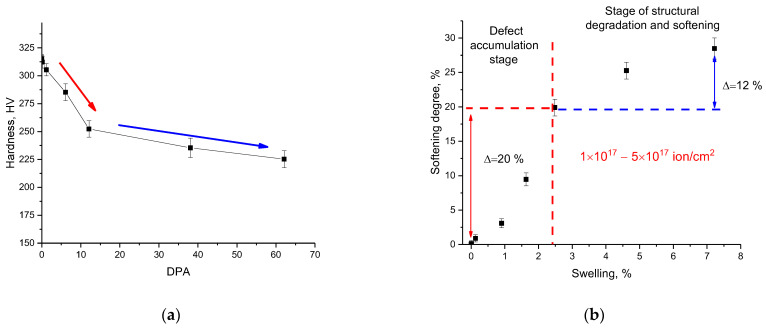
(**a**) Results of changes in the microhardness value depending on the value of atomic displacements; (**b**) results of the near-surface layer softening depending on the swelling degree.

Figure 4a shows the changes in the microhardness of the studied samples depending on the dose of accumulated atomic displacements during irradiation of a near-surface layer 200–300 nm thick. As can be seen from the data presented, the greatest changes in hardness occur when the value of atomic displacements changes in the range from 0.01 to 10 dpa, for which a significant decrease in hardness values is observed, which is more than 20% of the initial value. This behavior of changes in the hardness of the near-surface layer can be due to the accumulation of structural distortions and deformations. 

The increase in these contributes to the formation of highly distorted metastable regions in the near-surface layer structure, resulting in its softening and destabilization. At the same time, an assessment of the dependence of the softening degree on swelling (see Figure 4b) showed that at small values of atomic displacements, characterized by the radiation damage accumulation in the form of point defects and interstitial atoms, as well as their accumulation in the near-surface layer, leads to a sharp decrease in hardening, while for the stage with a characteristic dominance of the effect of swelling due to the formation of gas-filled regions, the change in strength properties is less pronounced (∆SD = 12% for the range of swelling 3–7%, while for the range of swelling <3% ∆SD = 20%). The effect of self-hardening can explain this difference as a result of the formation of highly distorted or amorphous-like inclusions in the damaged layer structure, the presence of which leads to a slight increase in the resistance of the damaged material to external influences. Furthermore, one of the explanations for this effect may be that with an increase in the implanted helium concentration at radiation fluences above 10^17^ ion/cm^2^, the effects associated with the formation of gas-filled bubbles and subsequent swelling begin to dominate in structural changes in the near-surface damaged layer, which partially reduces the concentration of deformation inclusions due to their suppression by the formed gas-filled bubbles.

**Figure 5:**
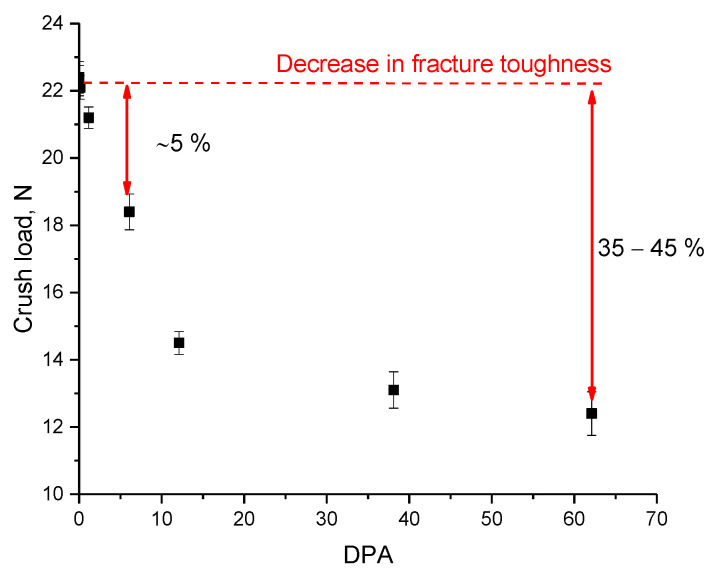
Results of changes in crack resistance depending on the value of atomic displacements.

Figure 5 shows the results of the determination of the crack resistance during a single compression of samples exposed to irradiation. According to the data obtained, the crack resistance is preserved at small atomic displacements, which are characterized by the dominance of the formation of point defects and their accumulation. In the case when swelling processes begin to dominate in the structure, crack resistance is significantly reduced, which may be due to processes associated with the formation of gas-filled bubbles, leading to accelerated propagation of microcracks with an increase in the volume of filled cavities, thereby increasing the degree of stress and increasing the likelihood of embrittlement or cracking under external influences.

**Figure 6:**
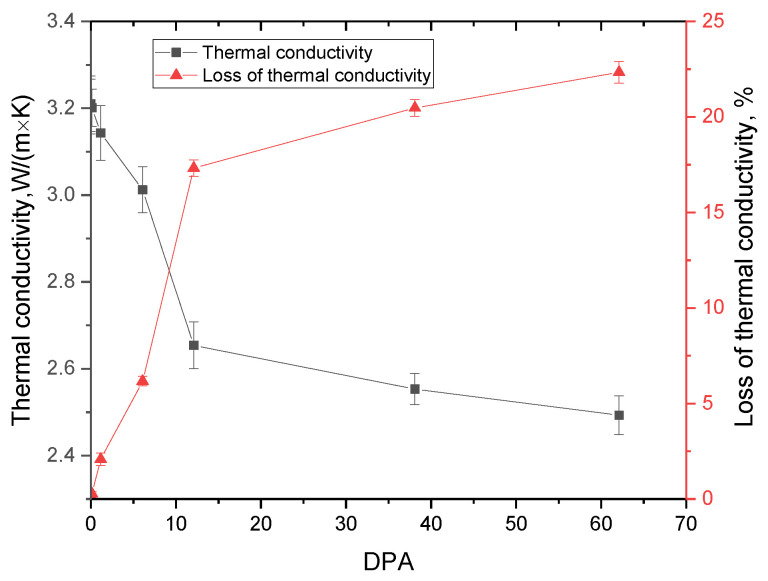
Results of changes in thermophysical properties depending on the accumulated atomic displacements.

Figure 6 shows the data on changes in the values of the thermophysical parameters of the ceramics under study depending on the accumulated value of atomic displacements in the near-surface layer, which reflects the deterioration of the thermal conductivity of the ceramics. The effect of reduction in the thermal conductivity decrease and an increase in heat losses at high fluences and atomic displacements was also established in [43,44] when the ceramic materials were irradiated with neutron radiation.

As is known, due to low electron density and dielectric nature in ceramics, the main mechanisms of heat transfer are due to phonons. In this case, the phonon relaxation time in the case of a change in the concentration of point defects is proportional to the concentration of defects in the structure associated with the formation of vacancies, as well as a change in the volume of the crystal lattice. In the case of the formation of defects of a more complex configuration in the form of dislocation loops or clusters, the phonon relaxation time is proportional to the density of dislocation loops, which makes the processes of heat transfer with the help of phonons strongly dependent on the concentration and types of defects in the structure of the damaged material. In view of this, the change in thermal conductivity has a strongly pronounced dependence on the dominance of types of defects during their accumulation and subsequent evolution. Therefore, when an increase in vacancy and point defects dominates in the structure of the damaged layer, the processes of heat transfer due to phonons are greatly hindered. This leads to a sharp deterioration in thermal conductivity and an increase in the value of heat losses in the damaged layer. It can result in overheated regions in the structure of dispersed fuel near the interface between the fissile material and the damaged layer of the inert matrix. However, in the case when the gas swelling processes begin to dominate in the structural degradation of the near-surface layer, as well as the formation of more complex defective inclusions in the form of dislocation loops or regions of disorder, the decrease in thermal conductivity is much lower with an increase in the accumulated dose of atomic displacements.

**Figure 7:**
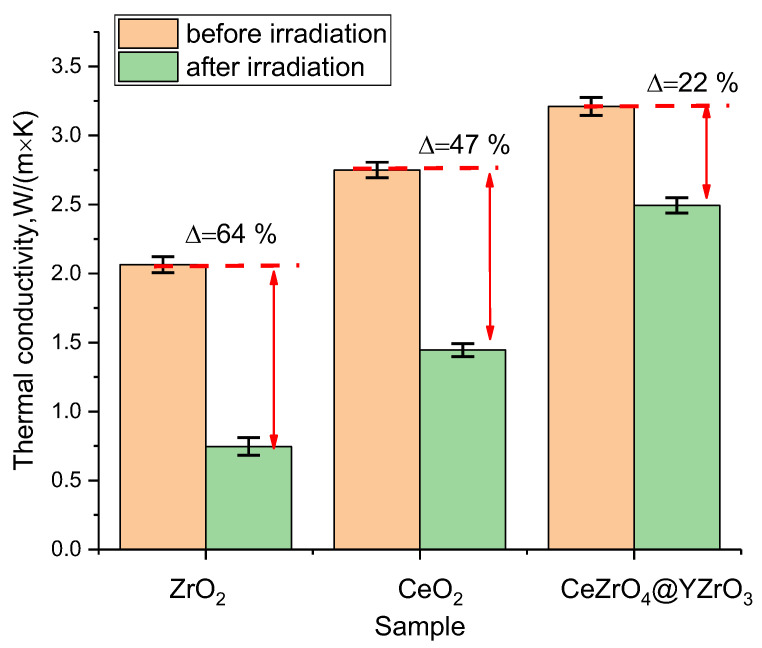
Comparative analysis of thermophysical parameters of various types of ceramics after irradiation.

Figure 7 presents the results of a comparative analysis of changes in the strength and thermophysical parameters of the synthesized ceramics subjected to irradiation with the results of similar experiments on samples of single-phase ceramics based on ZrO_2_, CeO_2_ subjected to irradiation with low-energy He^2+^ ions at a fluence of 5 × 10^17^ ion/cm^2^.

As can be seen from the data presented, a change in the composition of ceramics from single-phase ZrO_2_ or CeO_2_ to two-phase CeZrO_4_–YZrO_3_ leads to an increase in thermal conductivity from 2.02–2.75 W/m×K to 3.2 W/m×K, which indicates an improvement in thermophysical parameters due to a change in the phase composition of ceramics. At the same time, as can be seen from evaluation results of the change in the thermal conductivity coefficient for the samples under study after irradiation, the maximum decrease of more than 60% was observed for ZrO_2_, for which, as is known from several works [29,30], processes of polymorphic transformations of the t-ZrO_2_ → c-ZrO_2_ type are observed during irradiation, which are accompanied by a change in thermophysical and strength parameters. At the same time, comparing the results of changes in the thermal conductivity of ZrO_2_ and CeZrO_4_–YZrO_3_ samples, it was found that in the case of CeZrO_4_–YZrO_3_ ceramics, the presence of two-phase leads to a threefold increase in resistance to a decrease in thermophysical parameters, which indicates excellent prospects for the use of these types of ceramics as inert matrix materials.

## 4. Conclusions

This work is devoted to the study of the resistance of CeZrO_4_–YZrO_3_ ceramics to irradiation with He^2+^ ions in the dose range of 10^14^–5 × 10^17^ ion/cm^2^, the characteristic concentration of atomic displacements from 0.01 to 60 dpa and the concentration of implanted ions at the maximum penetration depth of more than 25%, according to the calculated data. To characterize the data obtained, the methods of X-ray diffraction analysis, indentation and single compression, and the determination of the thermal conductivity coefficient, were used. During the analysis of the structural parameters and their changes depending on the irradiation fluence, it was found that the main structural damage mechanisms at low atomic displacements are the formation of point defects and deformational distortion of the crystal lattice by the stretching mechanism, while at high atomic displacements, the dominant structural damage mechanisms are associated with the accumulation of implanted helium and subsequent swelling. It was established that when swelling processes dominate in the damaged layer structure at high irradiation fluences, the degree of change in softening and deterioration of thermophysical parameters is much lower than in the case of dominance of the formation of vacancy and point defects at low irradiation fluences.

## Data Availability

Not applicable.

## References

[1] Lambert T., Nghiem X.U. (2014). Review of the deployment of and research into generation III & IV nuclear fission reactors for power generation. PAM Rev. Energy Sci. Technol..

[2] Van Goethem G. (2011). Nuclear fission, today and tomorrow: From renaissance to technological breakthrough (Generation IV). J. Press. Vessel Technol..

[3] Romanello V., Salvatores M., Gabrielli F., Vezzoni B., Maschek W., Schwenk-Ferrero A., Petrovic B. (2012). Comparison of the waste transmutation potential of different innovative dedicated systems and impact on the fuel cycle. Fusion Sci. Technol..

[4] Kong L., Wei T., Zhang Y., Karatchevtseva I., Chironi I. (2020). One-pot synthesis of Ln2Sn2O7 pyrochlore and MgAl2O4 spinel by soft chemistry route as potential inert matrix fuel system, and the microstructural analysis. J. Nucl. Mater..

[5] Pillon S., Wallenius J. (2006). Oxide and nitride TRU fuels: Lessons drawn from the CONFIRM and FUTURE projects of the 5th European Framework Program. Nucl. Sci. Eng..

[6] Costantini J.M., Gutierrez G., Lelong G., Guillaumet M., Rahman M.M., Yasuda K. (2022). Raman spectroscopy study of damage in swift heavy ion-irradiated ceramics. J. Raman Spectrosc..

[7] Abrinaei F., Aghabeygi S. (2022). Optimization on preparation conditions to improve the nonlinear optical response of ZnO/TiO_2_/ZrO_2_ ternary nanocomposites under continuous-wave laser irradiation. Optik.

[8] Mistarihi Q., Umer M.A., Kim J.H., Hong S.H., Ryu H.J. (2015). Fabrication of ZrO_2_-based nanocomposites for transuranic element-burning inert matrix fuel. Nucl. Eng. Technol..

[9] Kursoglu P., Motro P.F.K., Yurdaguven H. (2013). Shear bond strength of resin cement to an acid etched and a laser irradiated ceramic surface. J. Adv. Prosthodont..

[10] Tan Y., Ma L., Akhmadaliev S., Zhou S., Chen F. (2016). Ion irradiated Er: YAG ceramic cladding waveguide amplifier in C and L bands. Opt. Mater. Express.

[11] Ba J., Zeng R., Yan X., Li R., Wu W., Li F., Xiang X., Meng D., Tang T. (2021). Long-term helium bubble evolution in sequential He^+^ and H^+^ irradiated Li_4_SiO_4_. Ceram. Int..

[12] Tynyshbayeva K.M., Kadyrzhanov K.K., Kozlovskiy A.L., Kuldeyev Y.I., Uglov V., Zdorovets M.V. (2022). Study of Helium Swelling and Embrittlement Mechanisms in SiC Ceramics. Crystals.

[13] Kislitsin S.B., Ryskulov A.E., Kozlovskiy A.L., Ivanov I.A., Uglov V.V., Zdorovets M.V. (2020). Degradation processes and helium swelling in beryllium oxide. Surf. Coat. Technol..

[14] Huang Z., Ma N., Qi J., Guo X., Yang M., Tang Z., Lu T. (2019). Defect-fluorite Gd_2_Zr_2_O_7_ ceramics under helium irradiation: Amorphization, cell volume expansion, and multi-stage bubble formation. J. Am. Ceram. Soc..

[15] Lin Z., Wu C., He H., Jiang S., Ren F., Cao L., Zhang J. (2021). In-situ transmission electron microscopy observation of the helium bubble evolution in pre-irradiated fluorapatite during annealing. Ceram. Int..

[16] Kozlovskiy A.L. (2021). Determination of critical doses of radiation damage to AlN ceramic under irradiation of helium and hydrogen ions. Eurasian Phys. Tech. J..

[17] Liu Y., Zhu Y., Shen T., Chai J., Niu L., Li S., Wang Z. (2021). Irradiation response of Al_2_O_3_-ZrO_2_ ceramic composite under He ion irradiation. J. Eur. Ceram. Soc..

[18] Sun J., You Y.W., Wu X., Song H.Y., Li B.S., Liu C.S., Krsjak V. (2022). Segregation and diffusion behaviours of helium at grain boundaries in silicon carbide ceramics: First-principles calculations and experimental investigations. J. Eur. Ceram. Soc..

[19] Su R., Zhang H., Shi L., Wen H. (2019). Formation of nanostructures in Ti_2_AlC induced by high-temperature helium irradiation. J. Eur. Ceram. Soc..

[20] Golovkina L.S., Orlova A.I., Nokhrin A.V., Boldin M.S., Chuvil’deev V.N., Sakharov N.V., Zelenov A.Y. (2018). Spark Plasma Sintering of fine-grain ceramic-metal composites based on garnet-structure oxide Y_2.5_Nd_0_._5_Al_5_O_12_ for inert matrix fuel. Mater. Chem. Phys..

[21] Ronchi C., Ottaviani J.P., Degueldre C., Calabrese R. (2003). Thermophysical properties of inert matrix fuels for actinide transmutation. J. Nucl. Mater..

[22] Hellwig C., Kasemeyer U. (2003). Inert matrix fuel performance during the first two irradiation cycles in a test reactor: Comparison with modelling results. J. Nucl. Mater..

[23] Frieß F., Liebert W. (2022). Inert-matrix fuel for transmutation: Selected mid-and long-term effects on reprocessing, fuel fabrication and inventory sent to final disposal. Prog. Nucl. Energy.

[24] Shelley A., Ovi M.H. (2022). Possibility of curium as a fuel for VVER-1200 reactor. Nucl. Eng. Technol..

[25] Bhandari K., Grover V., Roy A., Sahu M., Shukla R., Banerjee J. (2022). Nd^3+^-Y_3_Al_5_O_12_ system: Iso-valent substitution driven structural phase evolution and thermo-physical behavior. J. Mol. Struct..

[26] Kasemeyer U., Hellwig C., Lee Y.W., Ledergerber G., Sohn D.S., Gates G.A., Wiesenack W. (2001). The irradiation test of inert-matrix fuel in comparison to uranium plutonium mixed oxide fuel at the halden reactor. Prog. Nucl. Energy.

[27] Kumar R., Chauhan V., Gupta D., Upadhyay S., Ram J., Kumar S. (2021). Advancement of High–k ZrO_2_ for Potential Applications: A Review. Indian J. Pure Appl. Phys..

[28] Zhang J., Wang H., Wei H., Zhang J., Tang C., Lu C., Li Y. (2021). Modelling of effective irradiation swelling for inert matrix fuels. Nucl. Eng. Technol..

[29] Ghyngazov S.A., Boltueva V.A., O’Connell J.H., Vershinina T.N., Kirilkin N.S., Rymzhanov R.A., Surzhikov A.P. (2022). Swift heavy ion induced phase transformations in partially stabilized ZrO_2_. Radiat. Phys. Chem..

[30] Alin M., Kozlovskiy A.L., Zdorovets M.V., Uglov V.V. (2022). Study of the mechanisms of the t-ZrO_2_ → c-ZrO_2_ type polymorphic transformations in ceramics as a result of irradiation with heavy Xe^22+^ ions. Solid State Sci..

[31] Pu G., Zou J., Lin L., Zhang K., Liu B., Ma F., Li Q. (2019). Effects of He ion irradiation on the microstructures and mechanical properties of t’phase yttria-stabilized zirconia ceramics. J. Alloys Compd..

[32] Sattonnay G., Lahrichi M., Herbst-Ghysel M., Garrido F., Thomé L. (2007). Stress field induced by swift heavy ion irradiation in cubic yttria stabilized zirconia. J. Appl. Phys..

[33] Kalita P., Ghosh S., Sattonnay G., Singh U.B., Grover V., Shukla R., Avasthi D.K. (2017). Role of temperature in the radiation stability of yttria stabilized zirconia under swift heavy ion irradiation: A study from the perspective of nuclear reactor applications. J. Appl. Phys..

[34] Singh A., Saini R., Kumar P., Kandasami A. (2022). Tailoring of defects dependent magnetic properties of swift heavy ion irradiated CeO2 for spintronics application. J. Appl. Phys..

[35] Weber M.H., McCloy J.S., Halverson C.R., Karcher S.E., Mohun R., Corkhill C.L. (2022). Characterization of vacancy type defects in irradiated UO_2_ and CeO_2_. MRS Adv..

[36] Lan J., Zhai P., Nan S., Xu L., Niu J., Tian C., Ewing R.C. (2022). Phase stability of pre-irradiated CeO_2_ with swift heavy ions under high pressure up to 45 GPa. J. Am. Ceram. Soc..

[37] Liu P.P., Xue L.W., Yu L.P., Liu J.L., Hu W., Zhan Q., Wan F.R. (2019). Microstructure change and swelling of helium irradiated beryllium. Fusion Eng. Des..

[38] Su Q., Inoue S., Ishimaru M., Gigax J., Wang T., Ding H., Nastasi M. (2017). Helium irradiation and implantation effects on the structure of amorphous silicon oxycarbide. Sci. Rep..

[39] Krsjak V., Shen T., Degmova J., Sojak S., Korpas E., Noga P., Garner F.A. (2022). On the helium bubble swelling in nano-oxide dispersion-strengthened steels. J. Mater. Sci. Technol..

[40] Egeland G.W., Valdez J.A., Maloy S.A., McClellan K.J., Sickafus K.E., Bond G.M. (2013). Heavy-ion irradiation defect accumulation in ZrN characterized by TEM, GIXRD, nanoindentation, and helium desorption. J. Nucl. Mater..

[41] Zinkle S.J., Snead L.L. (2014). Designing radiation resistance in materials for fusion energy. Annu. Rev. Mater. Res.

[42] Singh B.N., Zinkle S.J. (1993). Defect accumulation in pure fcc metals in the transient regime: A review. J. Nucl. Mater..

[43] Nordlund K., Zinkle S.J., Sand A.E., Granberg F., Averback R.S., Stoller R.E., Simeone D. (2018). Primary radiation damage: A review of current understanding and models. J. Nucl. Mater..

[44] Snead L.L., Zinkle S.J., White D.P. (2005). Thermal conductivity degradation of ceramic materials due to low temperature, low dose neutron irradiation. J. Nucl. Mater..

